# Long-term outcomes of eltrombopag plus cyclosporine A in eltrombopag-exposed patients with glucocorticoid-resistant or -dependent primary immune thrombocytopenia: a prospective, multicenter, single-arm, phase II study with extended follow-up

**DOI:** 10.3389/fimmu.2026.1867103

**Published:** 2026-09-09

**Authors:** Yuhui Yuan, Jie Jin, Jin Lai, Changle Huang, Xuwei Shao, Fangqi Cheng, Huifang Jiang, Jiayue Qin, Lihong Cao

**Affiliations:** 1 Zhejiang Chinese Medical University, Hangzhou, Zhejiang, China; 2 Department of Hematology, The First Affiliated Hospital, Zhejiang University School of Medicine, Hangzhou, China; 3 Department of Hematology, Shulan (Hangzhou) Hospital Affiliated to Zhejiang Shuren University Shulan International Medical College, Hangzhou, China; 4 Department of Hematology, Tongde Hospital of Zhejiang Province, Hangzhou, China; 5 Department of Medical Affairs, Acornmed Biotechnology Co., Ltd., Beijing, China

**Keywords:** CsA, ELT, ITP, long-term follow-up, sustained remission

## Abstract

**Background:**

Primary immune thrombocytopenia (ITP) is an autoimmune disorder characterized by thrombocytopenia, and approximately 20-30% of patients are refractory to or dependent on first-line glucocorticoid therapy. Our previous prospective phase II study demonstrated promising short-term efficacy of eltrombopag (ELT) combined with cyclosporine A (CsA); however, the durability of response, long-term safety, and patterns of treatment failure remain incompletely characterized.

**Methods:**

We conducted a long-term follow-up analysis of patients enrolled in a multicenter, single-arm, prospective phase II trial (ChiCTR2000040991). In the original study, adult patients with primary ITP who were resistant to or dependent on glucocorticoids were enrolled between December 2020 and December 2023. All patients had previously received ELT monotherapy without an adequate or durable response; therefore, no ELT-naïve patients were included in this cohort. Patients received ELT (initial dose: 50 mg/day) combined with CsA (3 mg/kg/day, target trough concentration: 150-250 μg/L) for 8 weeks, and responders continued maintenance therapy. The follow-up period for the present analysis was extended until January 1, 2026. Safety and long-term Kaplan-Meier analyses included all 28 patients according to the intention-to-treat (ITT) principle, whereas the 8-week efficacy analysis included the 25 patients who completed 8 weeks of treatment. The three patients who discontinued within the first 8 weeks were included in the Kaplan-Meier analysis and counted as treatment-failure events at the time of discontinuation. The primary endpoints were the overall response rate (ORR) and complete response (CR) rate at 8 weeks, and the long-term sustained remission rate. Secondary endpoints included duration of response and the incidence of adverse events. Univariate logistic regression was performed as an exploratory analysis of associations between baseline variables and treatment failure.

**Results:**

A total of 28 patients were included, with a median follow-up of 32.46 months. Of these, 17 (60.7%) had shown previous ELT non-response and 11 (39.3%) had achieved a previous response followed by relapse. Previous ELT response status was not significantly associated with treatment failure (OR, 3.810; 95% CI, 0.738-19.663; *P* = 0.110). The ORR at week 8 was 92.0% (23/25), including a CR rate of 72.0% (18/25). The sustained remission rates at 12 and 24 months were 64.3% and 57.1%, respectively. Treatment failure occurred in 15 patients (53.6%). Adverse event (AE)-related permanent treatment discontinuation contributed to eight treatment-failure events, including two early discontinuations and six secondary relapses after discontinuation. Two patients who relapsed after discontinuation due to AEs achieved CR again after reinitiating the same regimen. AEs occurred in 24 patients (85.7%), and grade 3–5 AEs occurred in four patients (14.3%), including two fatal infections, corresponding to an infection-related mortality of 7.1% (2/28). No thrombotic events were observed. In exploratory univariate logistic regression, a higher baseline bleeding score was associated with treatment failure (OR per 1-point increase, 2.102; 95% CI, 1.066-4.146; *P* = 0.032); however, multivariable adjustment was not feasible because of the limited number of outcome events.

**Conclusions:**

ELT plus CsA demonstrated high early response rates and durable disease control in a subset of ELT-exposed patients with glucocorticoid-resistant or -dependent ITP. AE-related treatment discontinuation was an important contributor to treatment failure, and the occurrence of two fatal infections highlights the need for careful patient selection and close infection monitoring. The re-challenge and baseline bleeding-score findings were exploratory and require validation in larger, controlled studies.

## 1 Introduction

Primary immune thrombocytopenia (ITP) is an autoimmune disorder characterized by isolated thrombocytopenia, primarily resulting from increased platelet destruction and impaired platelet production (1). Glucocorticoids (GCs) are the first-line treatment for ITP; however, approximately 20-30% of adult patients progress to chronic or refractory disease after treatment (2), and these patients face an increased risk of bleeding and a significant impairment in health-related quality of life (3). For patients who are refractory to or dependent on GCs, second-line therapies are typically required, including thrombopoietin receptor agonists (TPO-RAs, e.g., eltrombopag (ELT)), anti-CD20 monoclonal antibodies, splenectomy, and various immunosuppressive agents (4).

ELT is an oral TPO-RA that promotes platelet production by stimulating megakaryocyte proliferation and differentiation (5). Cyclosporine A (CsA) is a calcineurin inhibitor that suppresses T-cell activation and the production of cytokines such as interleukin-2 (IL-2), thereby reducing immune-mediated platelet destruction (6). These two agents target two key pathogenic mechanisms of ITP—impaired platelet production and immune-mediated platelet destruction—and their combination may exert potential synergistic effects.

Our group previously conducted a multicenter, single-arm, prospective phase II trial (ChiCTR2000040991), in which ELT plus CsA achieved an (overall response rate) ORR of 92% at week 8 in patients with ELT-exposed glucocorticoid-resistant or -dependent ITP (7). However, the original report primarily focused on short-term efficacy and safety, whereas the durability of response, long-term safety, and patterns of treatment failure remained incompletely characterized. Therefore, we extended the follow-up of this cohort to January 1, 2026, to evaluate long-term efficacy and safety, characterize treatment-failure patterns, and explore associations between baseline factors and treatment outcomes.

## 2 Methods

### 2.1 Study design

This study was a long-term follow-up analysis of patients enrolled in a multicenter, single-arm, prospective phase II trial (ChiCTR2000040991). Patients were enrolled in the original study between December 2020 and December 2023, and the inclusion and exclusion criteria have been described in detail in a previous report (7). The key points are as follows: Eligible patients were aged 18–75 years, had a diagnosis of ITP according to the Chinese guidelines (2020 version) (8), were resistant to or dependent on GCs (as defined in the original study (7)), and had a baseline platelet count <30 × 10^9^/L on two separate occasions at least 3 days apart. Glucocorticoid resistance, defined as failure to achieve a platelet count ≥30 × 10^9^/L after standard-dose prednisone followed by one or two cycles of high-dose dexamethasone; and glucocorticoid dependence, defined as inability to maintain a platelet count ≥30 × 10^9^/L during tapering to prednisone ≤5 mg/day or after discontinuation, or relapse after a repeated glucocorticoid course (7). Importantly, all patients enrolled in the original trial had previously received ELT monotherapy without an adequate or durable response; therefore, no ELT-naïve patients were included in the present cohort. Exclusion criteria included secondary ITP, active infections, malignancies, severe cardiac, hepatic, or renal dysfunction, and bone marrow dysplasia or fibrosis. All patients provided written informed consent, and the study protocol was approved by the institutional review boards of all participating centers. For the present long-term follow-up analysis, the data cutoff date was extended to January 1, 2026, and the analysis protocol was approved by the ethics committees with a waiver of additional informed consent, as only previously collected data were used.

### 2.2 Treatment and data collection

In the original study, ELT was administered at a protocol-specified initial dose of 50 mg once daily, and CsA was initiated at 3 mg/kg/day, with a target trough concentration of 150-250 μg/L. The ELT starting dose was selected based on adult dosing experience summarized in the updated international consensus report, which described initial doses of 25 or 50 mg/day and noted that 50 mg/day had been used in several studies of chronic ITP (9). All participants had previously received ELT monotherapy without an adequate or durable response and had platelet counts <30 × 10^9^/L at enrollment. Therefore, at the time of protocol development, 50 mg/day was selected as a pragmatic, study-specific dose with the clinical aim of achieving a timely increase to a hemostatic platelet count in this heavily pretreated population. Platelet counts were monitored weekly, and the doses of ELT and CsA were adjusted according to a prespecified platelet-guided dose-adjustment algorithm.

If the platelet count remained <30 × 10^9^/L after 2 weeks, the ELT dose was increased to 75 mg/day. If the platelet count was ≥200 × 10^9^/L for 2 consecutive weeks, the ELT dose was reduced to 25 mg/day; if it remained ≥300 × 10^9^/L for 2 consecutive weeks, ELT was discontinued. If the platelet count remained ≥300 × 10^9^/L for an additional 2 consecutive weeks after ELT discontinuation, the CsA dose was reduced stepwise by 50 mg/day. If the platelet count subsequently decreased to <150 × 10^9^/L for 2 consecutive weeks, the CsA dose was gradually increased in patients whose CsA dose had previously been reduced, whereas ELT was resumed at 25 mg/day in patients whose CsA dose had not been reduced previously.

In the event of a severe infection, CsA was temporarily discontinued and resumed after adequate control of the infection. Patients whose CsA treatment was interrupted for more than 2 weeks were withdrawn from the original study. Patients who failed to achieve a response at week 8 (defined as a platelet count ≥30 × 10^9^/L without bleeding) or who subsequently experienced relapse (defined as a platelet count <30 × 10^9^/L on two occasions at least 24 hours apart or recurrent bleeding) were also withdrawn from the original study.

Patients who achieved a response after the initial 8-week treatment period continued maintenance therapy with ELT plus CsA at the doses administered at week 8. During the maintenance phase, the doses of both agents continued to be adjusted according to the same prespecified platelet-guided dose-adjustment algorithm, as described in the original report (7).

For this long-term follow-up analysis, additional data up to January 1, 2026 were collected from the hospital electronic medical record system and outpatient follow-up records. Previous treatment exposure included GCs, thrombopoietin receptor agonists, rituximab, splenectomy, and immunosuppressive agents. Previous ELT response status was determined from the available pretreatment medical records. Patients were categorized as having previous ELT non-response or a previous response followed by relapse. Previous ELT non-response was defined as failure to achieve a response during ELT monotherapy, whereas previous response followed by relapse was defined as achievement of a response during ELT monotherapy followed by subsequent loss of response before enrollment. Treatment and follow-up data included dose adjustments, platelet counts, bleeding events, adverse events (AEs), and their management. Baseline bleeding severity was assessed using the bleeding score for adult primary ITP recommended in the Chinese guideline for the diagnosis and management of adult primary ITP (2020 version) (8). The score comprises an age component and a bleeding-symptom component, and the total score is calculated as the sum of the age score and the highest severity score among cutaneous, mucosal, and deep-organ bleeding manifestations. Adverse events were graded according to the Common Terminology Criteria for Adverse Events (CTCAE), version 5.0 (10); when no exact CTCAE preferred term was available, the corresponding “Other, specify” category was used. Outcome data included response status—defined as complete response (CR), platelet count ≥100 × 10^9^/L without bleeding, and response (R), platelet count ≥30 × 10^9^/L and at least a twofold increase from baseline without bleeding (11)—as well as time to relapse and final outcomes. Baseline exploratory biomarkers included platelet-associated immunoglobulin G (PAIgG), thrombopoietin (TPO), immunoglobulin G (IgG), complement component 3 (C3), natural killer (NK) cells, T-helper cell subsets (Th1, Th2, and Th17), and regulatory T cells (Tregs), including activated Tregs.

### 2.3 Study endpoints

The primary endpoints were the ORR and CR rates at 8 weeks, as well as the long-term sustained remission rate. Long-term sustained remission was defined as maintenance of CR or R without treatment failure up to the data cutoff date. Treatment failure was defined as primary non-response at week 8, subsequent relapse, or unplanned permanent discontinuation of the ELT plus CsA regimen because of AEs, patient-related factors, or other non-protocol reasons, including discontinuation while an ongoing response was maintained. Protocol-guided dose tapering or treatment discontinuation after sustained remission was not classified as treatment failure. Secondary endpoints included median time to response, duration of response (estimated using the Kaplan-Meier method), and the incidence, type, and severity of AEs, including grade 3–5 and fatal events. Exploratory endpoints included the assessment of associations between baseline clinical characteristics (e.g., age, disease duration, number of prior treatment lines, bleeding score, and platelet count) and immunological parameters with treatment outcomes.

### 2.4 Statistical analysis

Categorical variables were described as frequencies (percentages). Continuous variables with a normal distribution were presented as mean ± standard deviation (SD) and compared using the independent samples t-test, whereas non-normally distributed variables were expressed as median (interquartile range, IQR) and compared using the Mann-Whitney U test. Comparisons of categorical variables were performed using the chi-square test or Fisher’s exact test.

The 8-week efficacy analysis included the 25 patients who completed 8 weeks of treatments. The safety analysis included all 28 patients who received at least one dose of the combination therapy, whereas the long-term Kaplan-Meier analysis followed the ITT principle and included all 28 patients. In the Kaplan-Meier analysis, the three patients who discontinued treatment within the first 8 weeks were counted as treatment-failure events at the time of discontinuation. The Kaplan-Meier method was used to estimate sustained remission. Time was calculated from treatment initiation to the composite treatment-failure endpoint defined in Section 2.3 or censoring. Patients who remained in sustained remission at the data cutoff were censored on that date.

Univariate logistic regression was used to explore associations between baseline variables, including previous ELT response status, and the composite treatment-failure endpoint. Multivariable logistic regression was not performed because of the limited number of outcome events. These association analyses were considered exploratory. All analyses were performed using SPSS version 27.0 (IBM Corp., Armonk, NY, USA), and a two-sided P value <0.05 was considered statistically significant.

## 3 Results

### 3.1 Patient baseline characteristics

Baseline characteristics of the 28 patients stratified by final treatment outcome are summarized in **Table 1**. The median age was 42.5 years (range, 18-74), and 13 patients (46.4%) were male. The median disease duration was 17.15 months (range, 3–279), and the median number of prior treatment lines was 2 (range, 2–5). The median baseline platelet count was 8.0 × 10^9^/L (range, 1–28), and the median bleeding score was 2 (range, 0–6). All 28 patients had previously received GCs and ELT. Based on their previous response to ELT monotherapy, 17 patients were classified as having previous ELT non-response, whereas 11 had achieved a previous response followed by relapse. Twenty-two patients (78.6%) had received intravenous immunoglobulin, five (17.9%) recombinant human thrombopoietin, one (3.6%) rituximab, and two (7.1%) had undergone splenectomy. Seven patients (25.0%) had previously received at least one immunosuppressive agent, including cyclosporine A, tacrolimus, or sirolimus. Because prior ELT exposure was universal, outcomes could not be compared between TPO-RA-naive and TPO-RA-exposed subgroups.

**Table 1:** Baseline characteristics stratified by final treatment outcome.

Variable	Overall (n = 28)	Treatment failure group (n = 15)	Treatment success group (n = 13)	*P* value
Age (years)	42.50 (29.25-53.00)	32.00 (29.00-54.00)	43.00 (34.50-52.50)	0.896
Sex (male), n (%)	13 (46.40%)	7 (46.70%)	6 (46.20%)	1.000
Disease duration (months)	17.20 (9.80-51.00)	27.00 (12.00-111.00)	15.00 (6.00-33.00)	0.146
Baseline bleeding score	1.82 ± 1.58	2.47 ± 1.64	1.08 ± 1.19	0.018
Number of prior treatment lines	2.00 (2.00-3.00)	2.00 (2.00-4.00)	2.00 (2.00-3.00)	0.214
Platelet count (× 10^9/L)	10.50 ± 8.19	7.80 ± 5.61	13.62 ± 9.73	0.073
PAIgG	38.09 ± 9.05	34.80 ± 9.28	41.64 ± 7.60	0.047
TPO (pg/mL)	215.17 ± 294.66	282.52 ± 400.78	142.65 ± 55.85	0.225
IgG (g/L)	13.53 ± 5.06	13.27 ± 5.33	14.00 ± 4.88	0.769
C3 (g/L)	0.94 ± 0.27	0.87 ± 0.28	1.09 ± 0.20	0.079
CD4+ T cells/CD8+ T cells ratio	1.31 (0.77-1.92)	1.11 (0.74-1.82)	1.31 (0.75-2.01)	0.771
Th1/Th2 ratio	0.31 (0.18-0.74)	0.49 (0.14-1.02)	0.31 (0.21-0.62)	0.961
Th17/Treg ratio	2.08 ± 1.49	1.81 ± 1.27	2.38 ± 1.70	0.338
Th17/activated Treg ratio	6.13 (3.87-10.67)	5.07 (1.53-8.50)	9.26 (4.97-14.00)	0.089
Activated Treg/total Treg ratio	0.31 (0.17-0.51)	0.35 (0.16-0.56)	0.27 (0.16-0.38)	0.264
Previous ELT response status, n (%)				0.137
Previous ELT non-response	17 (60.7%)	7 (46.7%)	10 (76.9%)	
Previous response followed by relapse	11 (39.3%)	8 (53.3%)	3 (23.1%)	

Continuous variables are presented as mean ± SD or median (IQR), and categorical variables as counts (%). Normally distributed continuous variables were compared using the independent-samples t test, whereas non-normally distributed continuous variables were compared using the Mann-Whitney U test. Categorical variables were compared using the chi-square test or Fisher’s exact test, as appropriate. P values represent comparisons between the treatment-failure and treatment-success groups. The comparison of previous ELT response status between the treatment-failure and treatment-success groups was performed using Fisher’s exact test. PAIgG, platelet-associated immunoglobulin G; TPO, thrombopoietin; IgG, immunoglobulin G; C3, complement component 3; Th1, type 1 helper T cells; Th2, type 2 helper T cells; Th17, type 17 helper T cells; Treg, regulatory T cells.

Compared with the treatment success group, the treatment failure group had a significantly higher bleeding score (2.47 ± 1.64 vs. 1.08 ± 1.19, *P* = 0.018). A statistically significant difference was also observed in PAIgG levels (*P* = 0.047). Platelet count (*P* = 0.073), C3 levels (*P* = 0.079), and the Th17/activated Treg ratio (P = 0.089) showed trends toward differences between groups. Age, sex, disease duration, number of prior treatment lines, TPO levels, IgG levels, and other T-cell subsets were similarly distributed between the two groups (all *P* > 0.05).

### 3.2 Efficacy outcomes

Of the 28 patients, 25 completed at least 8 weeks of treatment. Three patients discontinued the study regimen within the first 8 weeks, including two because of early AEs and one for a reason unrelated to AEs. The long-term Kaplan-Meier analysis followed the ITT principle and included all 28 patients. All three early discontinuations were counted as treatment-failure events at the time of discontinuation and were not excluded or censored. In contrast, the 8-week ORR and CR analyses were restricted to the 25 patients who completed 8 weeks of treatment. The ORR at 8 weeks was 92.0% (23/25), with a CR rate of 72.0% (18/25).

**Figure 1:**
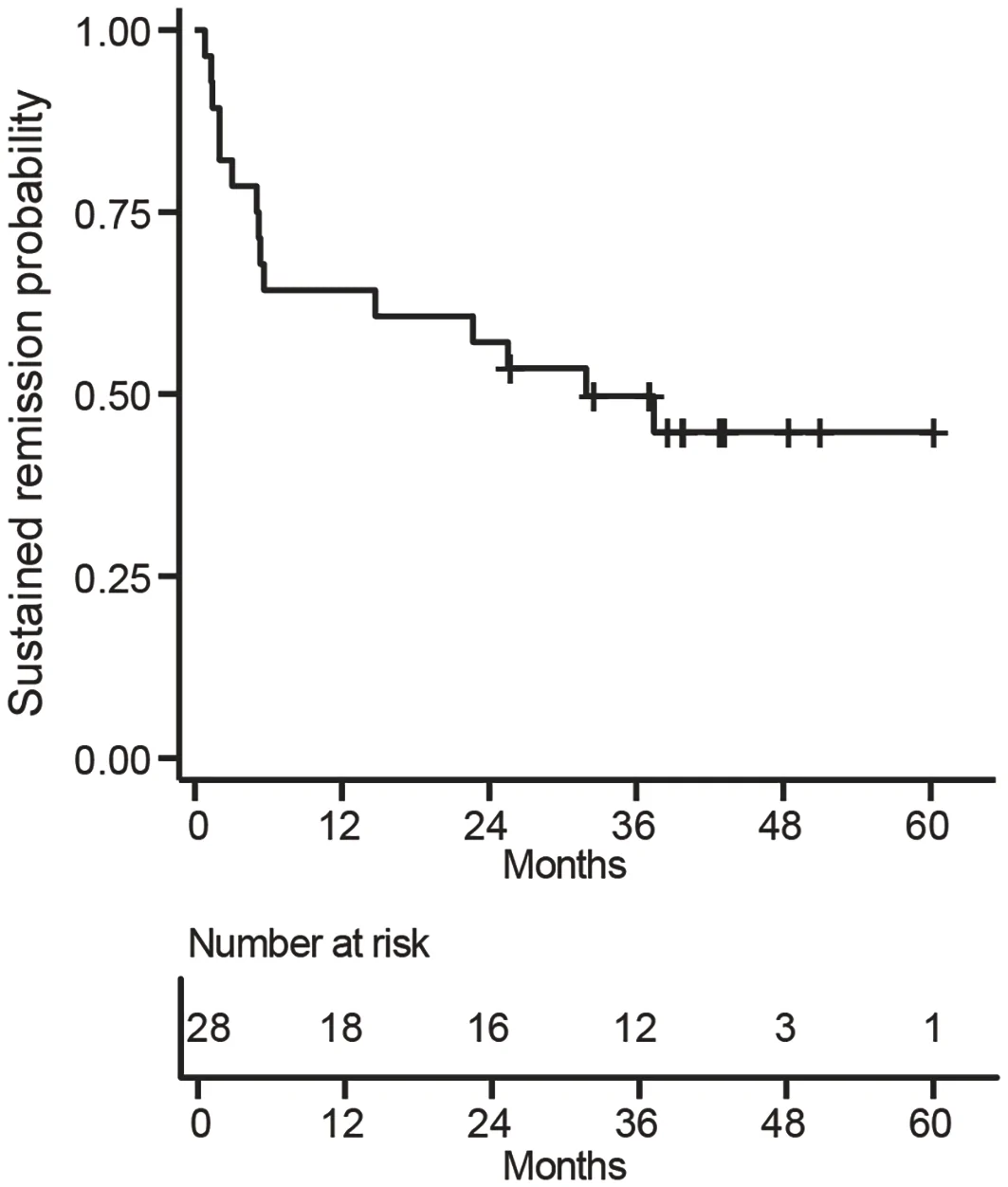
Kaplan-Meier curve of sustained remission in the intention-to-treat population. All 28 patients who received ELT plus CsA were included in the analysis. The three patients who discontinued the study regimen within the first 8 weeks were counted as treatment-failure events at the time of discontinuation and were not excluded or censored. The 12- and 24-month sustained remission rates were 64.3% and 57.1%, respectively. Sustained remission was defined as maintenance of complete response or response without treatment failure up to the data cutoff date. ELT, eltrombopag; CsA, cyclosporine A; ITP, immune thrombocytopenia.

As of January 1, 2026, the median follow-up duration was 32.46 months. Kaplan-Meier analysis showed that the 12- and 24-month sustained remission rates were 64.3% and 57.1%, respectively (**Figure 1**). At the end of follow-up, 13 patients maintained sustained remission.

### 3.3 Treatment failure patterns and retreatment outcomes

Of the 28 patients included in the ITT analysis, 15 (53.6%) met the study definition of treatment failure. These included three patients who discontinued the study regimen within the first 8 weeks, two patients with primary non-response at week 8, eight patients who subsequently experienced relapse, and two patients who permanently self-discontinued the study regimen despite maintaining a response. The latter two patients were classified as treatment failures because they permanently discontinued one component of the combination regimen for non-protocol reasons while still in response. One patient discontinued ELT on July 22, 2024, because of pregnancy planning. Her platelet count was 71 × 10^9^/L at the last available assessment on August 12, 2025, confirming maintenance of response after ELT discontinuation. The other patient discontinued CsA because of limited local availability and the high out-of-pocket cost, but continued ELT monotherapy and underwent follow-up at a local hospital. According to follow-up communication, the patient reported continued clinical benefit without loss of response, although the corresponding platelet-count records were unavailable for verification. Among the three early discontinuations, two were related to AEs and one occurred for a reason unrelated to AEs.

Among the eight patients with secondary relapse, six relapsed after permanent treatment discontinuation associated with AEs, whereas two relapsed following self-discontinuation of therapy. The types and CTCAE grades of the AEs leading to treatment discontinuation are detailed in the Safety analysis section. The treatment status at the time of secondary relapse was reviewed for all eight patients. CsA had been discontinued before relapse in all eight patients, whereas ELT was continued. Seven patients were receiving ELT at 50 mg once daily at relapse, and one was receiving ELT at 25 mg once daily. In one patient with persistent gingival hyperplasia, the CsA dose was initially reduced from 100 mg every 12 h to 50 mg every 12 h and was subsequently discontinued; relapse occurred 125 days after CsA discontinuation while ELT was continued at 50 mg once daily. In another patient, CsA had been gradually reduced to 10 mg every 12 h and maintained at this dose for approximately 1 year before the patient self-discontinued CsA; relapse occurred approximately 6 months later while ELT was continued at 25 mg once daily. One patient self-discontinued CsA because of limited local availability and relapsed 3 months later while continuing ELT at 50 mg once daily. Two patients discontinued CsA because of infection, experienced secondary relapse after CsA discontinuation while continuing ELT at 50 mg once daily, and subsequently died from the respective infections. Another patient discontinued CsA because of infection and relapsed shortly thereafter while continuing ELT at 50 mg once daily. Two additional patients discontinued CsA because of AEs and relapsed approximately 2 months and 1 month later, respectively, while continuing ELT at 50 mg once daily. Of the six patients who relapsed after AE-related permanent discontinuation of the study regimen, two were subsequently re-challenged during follow-up after resolution of the AEs, and both again achieved and maintained CR.

### 3.4 Safety analysis

The overall incidence of AEs was 85.7% (24/28). The most frequent AEs were gingival hyperplasia and hypertrichosis. Gingival hyperplasia occurred in 17 patients (60.7%), including 15 grade 1, one grade 2, and one grade 3 event. Hypertrichosis occurred in 10 patients (35.7%), all of whom had grade 1 events. Gastrointestinal discomfort occurred in four patients (14.3%), including two grade 1, one grade 2, and one grade 3 event. Infections occurred in three patients (10.7%), including one grade 2 and two grade 5 events. Other AEs included grade 1 palpitations, grade 2 amenorrhea, grade 1 knee joint swelling, grade 2 fatigue, and grade 1 hypertension, each occurring in one patient.

Eight patients discontinued the study regimen because of AEs. Two patients discontinued during the initial 8-week treatment period: one experienced grade 2 fatigue accompanied by grade 2 gastrointestinal discomfort, and the other experienced grade 3 gastrointestinal discomfort. Six patients permanently discontinued treatment after achieving an initial response. One patient discontinued because of grade 3 gingival hyperplasia requiring surgical intervention. Two patients developed infections during combination therapy that were ultimately fatal (grade 5), one of whom also had grade 1 gastrointestinal discomfort. One patient discontinued in the setting of grade 2 infection accompanied by grade 1 gingival hyperplasia; one discontinued in association with grade 1 hypertrichosis; and one discontinued in the setting of grade 2 gingival hyperplasia accompanied by grade 1 gastrointestinal discomfort. Because multiple concurrent AEs were documented in some patients, the relevant AEs associated with treatment discontinuation are reported together rather than assigning discontinuation to a single event.

Overall, grade 3–5 AEs occurred in four patients (14.3%), including two grade 3 events and two fatal infections. The two fatal infections corresponded to an infection-related mortality of 7.1% (2/28). Both fatal infections developed in 74-year-old patients during treatment with ELT plus CsA: one patient with diabetes developed cryptococcal meningitis complicated by pneumonia, and the other developed COVID-19 pneumonia. No thrombotic events were reported; however, the small cohort does not exclude thrombotic risk (9).

### 3.5 Exploratory univariate analysis of factors associated with treatment failure

In exploratory univariate logistic regression analysis (**Table 2**), a higher baseline bleeding score was associated with treatment failure (OR per 1-point increase, 2.102; 95% CI, 1.066-4.146; *P* = 0.032). Baseline platelet count (OR = 0.906, *P* = 0.071) and PAIgG (OR = 0.907, *P* = 0.059) showed nonsignificant trends toward associations with treatment failure. No statistically significant associations were observed for age, disease duration, number of prior treatment lines, TPO, IgG, C3, or T-cell subsets (all *P* > 0.05). Previous ELT response status was not significantly associated with treatment failure in univariate logistic regression. Treatment failure occurred in 7 of 17 patients (41.2%) with previous ELT non-response and in 8 of 11 patients (72.7%) with a previous response followed by relapse (OR for previous response followed by relapse versus previous non-response, 3.810; 95% CI, 0.738-19.663; *P* = 0.110).

**Table 2:** Univariate logistic regression analysis of factors associated with treatment failure.

Variable	OR	*P* value	95% CI
Age (per 1-year increase)	0.995	0.832	0.952-1.040
Disease duration (per 1-month increase)	1.012	0.192	0.994-1.031
Baseline bleeding score (per 1-point increase)	2.102	0.032	1.066-4.146
Number of prior treatment lines (per 1-line increase)	2.361	0.136	0.763-7.301
Platelet count (per 1 × 10^9^/L increase)	0.906	0.071	0.815-1.008
PAIgG (per 1-unit increase)	0.907	0.059	0.820-1.004
TPO (per 1-unit increase)	1.006	0.265	0.996-1.016
IgG (per 1 g/L increase)	0.971	0.755	0.807-1.168
C3 (per 1 g/L increase)	0.020	0.098	0.000-2.042
CD4^+^ T cells/CD8^+^ T cells ratio (per 1-unit increase)	0.671	0.363	0.284-1.586
Th1/Th2 ratio (per 1-unit increase)	2.526	0.276	0.477-13.373
Th17/Treg ratio (per 1-unit increase)	0.752	0.342	0.417-1.355
Th17/activated Treg ratio (per 1-unit increase)	0.909	0.169	0.793-1.042
Activated Treg/total Treg ratio (per 1-unit increase)	1.016	0.967	0.483-2.138
Previous ELT response status (response followed by relapse vs. non-response)	3.810	0.110	0.738-19.663

For continuous variables, OR represents the change in odds of treatment failure per one-unit increase in the corresponding variable. Previous ELT non-response was used as the reference category. PAIgG, platelet-associated immunoglobulin G; TPO, thrombopoietin; IgG, immunoglobulin G; C3, complement component 3; Th1, type 1 helper T cells; Th2, type 2 helper T cells; Th17, type 17 helper T cells; Treg, regulatory T cells.

## 4 Discussion

In this prospective phase II cohort of ELT-exposed patients with extended follow-up, ELT plus CsA produced high early response rates and durable disease control in a subset of patients with glucocorticoid-resistant or -dependent ITP. AE-related treatment discontinuation accounted for an important proportion of treatment failures, while severe infection emerged as the principal safety concern. Two patients regained CR after re-challenge, although these isolated observations do not establish that treatment interruption caused the preceding relapse.

In recent years, thrombopoietin receptor agonists (TPO-RAs) have substantially expanded the treatment options for patients with persistent or chronic ITP. Real-world data have demonstrated substantial clinical activity of TPO-RAs in routine practice, with the TRAIT study reporting that 89% of adult patients with ITP achieved a platelet count ≥30 × 10^9^/L during treatment with their first TPO-RA (12). More specifically, Historical TPO-RA monotherapy studies provide relevant context for interpreting the early and long-term outcomes observed in the present study. In a multicenter, randomized phase III trial involving previously treated Chinese adults with chronic ITP, ELT monotherapy resulted in 57.7% of patients achieving a platelet count ≥50 × 10^9^/L during the first 6 weeks, whereas the ORR in our cohort was 92.0% at week 8 (13). However, the early response rates are not directly comparable because of differences in disease phase, treatment regimen, ELT starting dose, assessment time point, and response definition. Specifically, the historical study reported the proportion of patients achieving a platelet count ≥50 × 10^9^/L, whereas our ORR definition required a platelet count ≥30 × 10^9^/L, at least a twofold increase from baseline, and the absence of bleeding. In the long-term EXTEND study, which included 302 adults with persistent or chronic ITP and a median ELT exposure of 2.37 years, 85.8% of patients achieved a platelet response at least once, and 52% of evaluable patients maintained a continuous response for at least 25 weeks. In our cohort, the sustained remission rates at 12 and 24 months were 64.3% and 57.1%, respectively (14). The 24-month sustained remission rate was numerically close to the continuous-response proportion reported in EXTEND, indicating that disease control was maintained in a substantial proportion of patients receiving ELT plus CsA. Nevertheless, this comparison is descriptive because continuous response for at least 25 weeks in EXTEND is not equivalent to sustained remission at 24 months without treatment failure or a change in treatment regimen. Differences in eligibility criteria, previous treatment exposure, response definitions, concomitant therapy, and censoring rules further preclude direct comparison. Therefore, these historical data provide clinical context but do not establish superiority or synergistic efficacy of ELT plus CsA over ELT monotherapy. Controlled comparative studies are required to determine whether the addition of CsA provides incremental benefit over ELT monotherapy. Safety remains an important consideration for this regimen. Although gingival hyperplasia and hypertrichosis were the most frequent AEs and were predominantly grade 1-2, four patients experienced grade 3–5 AEs, including two fatal infections, corresponding to an infection-related mortality of 7.1% in this small cohort. The occurrence of these fatal infections indicates that the safety profile should be interpreted with caution and warrants particular attention when considering this regimen for older patients or those with diabetes or other comorbidities that may increase susceptibility to infection. Although a direct causal relationship with the combination regimen cannot be established in this uncontrolled study, the immunosuppressive properties of CsA provide a biological rationale for concern regarding susceptibility to severe infection. Careful patient selection, individualized benefit-risk assessment, patient education, and close monitoring for severe or opportunistic infection are therefore warranted. The present cohort was too small to identify reliable patient-level risk factors for severe infectious complications. CsA exerts its immunosuppressive effects by inhibiting calcineurin-dependent T-cell activation and IL-2 production (6), providing a biological rationale for attenuating T-cell-mediated immune dysregulation in ITP. Reduction or discontinuation of CsA could therefore theoretically be followed by loss of immune control. In the present cohort, two patients regained CR after re-challenge following AE-related treatment discontinuation, raising the possibility that treatment sensitivity may be retained in selected cases. However, these two cases do not establish that relapse resulted from treatment interruption rather than acquired resistance, and natural disease fluctuation or other unmeasured factors cannot be excluded. The efficacy and safety of re-challenge therefore require further evaluation, and any decision to reintroduce the regimen should be individualized according to the previous toxicity and infectious risk.

In addition to promoting platelet production, TPO-RAs may also exert immunomodulatory effects. Zhang et al. (15) reported that responders to ELT exhibited distinct transcriptomic profiles and T-cell clonal characteristics, suggesting a potential role in modulating immune tolerance. However, because immune parameters were not assessed longitudinally in the present study, no conclusions can be drawn regarding the immunomodulatory effects of ELT or its potential interaction with CsA. Further mechanistic studies are needed to clarify these effects.

In exploratory univariate analysis, a higher baseline bleeding score was associated with treatment failure. Because this score incorporates both age and the site and severity of bleeding, the observed association may reflect a combination of hemorrhagic burden, age-related risk, and other unmeasured clinical factors that influenced treatment outcomes. However, because multivariable adjustment was not feasible, residual confounding cannot be excluded, and baseline bleeding score cannot be considered an independent predictor of treatment failure. This finding should be regarded as hypothesis-generating and requires validation in larger prospective cohorts. Previous ELT response status was not significantly associated with treatment failure, and the wide confidence interval precludes firm conclusions regarding its predictive value.

This study has several strengths. It was based on a prospective cohort with a relatively long follow-up duration and included a systematic characterization of treatment-failure patterns.

However, several limitations should be acknowledged. First, the small sample size and limited number of outcome events reduced statistical power, precluded reliable multivariable adjustment, limited the precision of estimates for rare outcomes. The selected study population also restricted the generalizability of the findings to the broader population of patients with ITP. The exploratory association analyses may therefore be susceptible to random variation and residual confounding. Second, the protocol-specified ELT starting dose of 50 mg/day was higher than the ancestry-adjusted recommended starting dose of 25 mg/day for East Asian patients. The findings should therefore not be directly extrapolated to ELT-naïve East Asian patients or to treatment strategies using a lower starting dose. Third, the absence of a control group precluded direct attribution of the outcomes to the combination regimen and definitive comparison with ELT monotherapy or other treatments. Finally, immunological parameters were assessed only at baseline and were not monitored longitudinally. Larger, adequately powered, controlled studies are required to validate the long-term efficacy, safety, and exploratory prognostic findings.

In conclusion, ELT plus CsA achieved high early response rates and durable disease control in a subset of ELT-exposed patients with glucocorticoid-resistant or -dependent ITP. AE-related treatment discontinuation was an important cause of treatment failure, and severe infections warrant careful patient selection and close monitoring. Larger controlled studies are required to confirm the long-term benefit-risk profile and the exploratory prognostic findings.

## Data Availability

The raw data supporting the conclusions of this article will be made available by the authors, without undue reservation.

## References

[1] Cooper N Ghanima W . Immune thrombocytopenia. N Engl J Med. (2019) 381:945–55. doi: 10.1056/NEJMcp1810479 31483965

[2] Moulis G Germain J Comont T Brun N Dingremont C Castel B . Newly diagnosed immune thrombocytopenia adults: Clinical epidemiology, exposure to treatments, and evolution. Results of the Carmen multicenter prospective cohort. Am J Hematol. (2017) 92:493–500. doi: 10.1002/ajh.24702 28240787

[3] Cooper N Kruse A Kruse C Watson S Morgan M Provan D . Immune thrombocytopenia (Itp) World Impact Survey (I-Wish): Impact of Itp on health-related quality of life. Am J Hematol. (2021) 96:199–207. doi: 10.1002/ajh.26036 33107998 PMC7898815

[4] Neunert C Terrell DR Arnold DM Buchanan G Cines DB Cooper N . American Society of Hematology 2019 guidelines for immune thrombocytopenia. Blood Adv. (2019) 3:3829–66. doi: 10.1182/bloodadvances.2019000966 31794604 PMC6963252

[5] Erickson-Miller CL Delorme E Tian SS Hopson CB Landis AJ Valoret EI . Preclinical activity of eltrombopag (Sb-497115), an oral, nonpeptide thrombopoietin receptor agonist. Stem Cells. (2009) 27:424–30. doi: 10.1634/stemcells.2008-0366 19038790 PMC2729672

[6] Kahan BD . Cyclosporine. N Engl J Med. (1989) 321:1725–38. doi: 10.1056/NEJM198912213212507 2687689

[7] Huang C Jin J Lai J Zhen W Fan C Fu G . Efficacy and safety of eltrombopag in combination with cyclosporine a for the treatment of adult refractory primary immune thrombocytopenia: A phase ii, multicenter, single-arm, prospective study. Clin Exp Med. (2026) 26:104. doi: 10.1007/s10238-025-01971-x 41498829 PMC12819436

[8] Thrombosis, Hemostasis Group CSoHCMA . Chinese guideline on the diagnosis and management of adult primary immune thrombocytopenia (Version 2020). Zhonghua Xue Ye Xue Za Zhi. (2020) 41:617–23. doi: 10.3760/cma.j.issn.0253-2727.2020.08.001 32942813 PMC7525165

[9] Provan D Arnold DM Bussel JB Chong BH Cooper N Gernsheimer T . Updated international consensus report on the investigation and management of primary immune thrombocytopenia. Blood Adv. (2019) 3:3780–817. doi: 10.1182/bloodadvances.2019000812 31770441 PMC6880896

[10] National Cancer Institute . Common Terminology Criteria for Adverse Events (CTCAE) Version 5.0. (2017).

[11] Rodeghiero F Stasi R Gernsheimer T Michel M Provan D Arnold DM . Standardization of terminology, definitions and outcome criteria in immune thrombocytopenic purpura of adults and children: Report from an international working group. Blood. (2009) 113:2386–93. doi: 10.1182/blood-2008-07-162503 19005182

[12] Cooper N Scully M Percy C Nicolson PLR Lowe G Bagot CN . Real-world use of thrombopoietin receptor agonists for the management of immune thrombocytopenia in adult patients in the United Kingdom: Results from the Trait study. Br J Haematol. (2024) 204:2442–52. doi: 10.1111/bjh.19345 38429869

[13] Yang R Li J Jin J Huang M Yu Z Xu X . Multicentre, randomised phase iii study of the efficacy and safety of eltrombopag in Chinese patients with chronic immune thrombocytopenia. Br J Haematol. (2017) 176:101–10. doi: 10.1111/bjh.14380 27734464

[14] Wong RSM Saleh MN Khelif A Salama A Portella MSO Burgess P . Safety and efficacy of long-term treatment of chronic/persistent Itp with eltrombopag: Final results of the Extend study. Blood. (2017) 130:2527–36. doi: 10.1182/blood-2017-04-748707 29042367

[15] Zhang H Zhang BM Guo X Xu L You X West RB . Blood transcriptome and clonal T-cell correlates of response and non-response to eltrombopag therapy in a cohort of patients with chronic immune thrombocytopenia. Haematologica. (2020) 105:e129–32. doi: 10.3324/haematol.2019.226688 31296576 PMC7049341

